# Identification of the IgG1 Induction Factor (Interleukin 4)

**DOI:** 10.3389/fimmu.2014.00628

**Published:** 2014-12-09

**Authors:** Eva Severinson

**Affiliations:** ^1^Department of Molecular Biosciences, The Wenner-Gren Institute, Stockholm University, Stockholm, Sweden

**Keywords:** interleukin 4, B lymphocytes, Ig class switching, molecular cloning, cell culture

This is an opinion based on the paper “Cloning of cDNA encoding the murine IgG1 induction factor by a novel strategy using SP6 promoter” (1).

The mechanism by which B cells can produce classes other than IgM and IgD has interested me since the middle of the 1970s. It was initially not clear that B cells could produce more than one Ig class, whereas we now know that they first produce IgM and IgD and then – after activation – they can switch to produce IgG, IgA, or IgE. Interleukin 4 (IL-4) can regulate Ig class switching in mice to IgG1 and IgE, and in humans to some subclasses of IgG and to IgE. Other cytokines can induce activation of various Ig classes and subclasses. Below is a personal tale of how I became involved in the molecular cloning of IL-4.

When I was a PhD student in the beginning of the 1970s, many of us at the Department of Immunobiology, Karolinska Institutet were intrigued by the question of whether, and if so how, B-cells switched Ig class. In the laboratory, Göran and Erna Möller and their groups were studying *in vitro* responses to T and B-cell mitogens, using mouse spleen cells. While stimulation with T cell-dependent antigens *in vivo* led to IgM and IgG responses, T cell-independent antigens did not induce an IgG response. Likewise, B-cell mitogens *in vitro* gave rise only to an IgM response. Since, we were concerned that fetal calf serum contained mitogenic substances; we used serum-free cultures. However, in order to obtain any response at all, we had to culture cells very densely. Kearny and Lawton then published a paper, describing the low-cell-density culture system (2). By culturing spleen cells at much lower cell densities, and by including fetal calf serum and 2-mercaptoethanol together with the mitogen lipopolysaccharide (LPS), B cells were induced to produce both IgM and IgG. This was an important discovery, since it was the first time IgG production had been induced *in vitro*. We tried to reproduce the experiment, but we had no good way to measure a polyclonal IgG response. At that time, we measured IgM responses in a plaque assay, using haptenated sheep erythrocytes, but it was not suitable for measuring IgG responses. Kearny and Lawton had used intracellular fluorescence in a UV microscope to detect IgG-producing cells, but we wanted a more quantitative assay. The idea occurred to me to develop an assay using protein A. It binds IgG and thus, by coating it to sheep erythrocytes, we might be able to develop a plaque assay for IgG-secreting cells. Antonio Coutinho and I decided to try the idea, so I went to visit him at Basel Institute for Immunology in Switzerland, where he worked as a postdoc. He thawed different mouse plasmacytomas that Fritz Melchers had in his lab. We could also use rabbit antisera to different mouse Ig isotypes, also from Fritz’s lab. I came to Basel in January 1976 and, if I remember correctly, the experiment worked first time. However, there were no plaques in the absence of developing antisera, as I had predicted. A likely explanation is that the rabbit IgG antisera bound the protein A and thus formed a bridge between protein A and the secreted Ig. This meant that we could detect any class of Ig from secreting cells if we just had a suitable antiserum. We worked day and night for 6 weeks to further develop the technique. More than once I met Susumi Tonegawa at the soft-drinks machine at night. He was then finishing his ground-breaking work on the movement of Ig V genes in B cell-development. A few months later, he presented his results at a Cold Spring Harbor meeting and I was in the audience. That was a very memorable moment. I left for the US 6 weeks later, and Antonio and Fritz finished the paper about the protein A plaque assay (3). Now, there was a good and convenient method to detect IgG-secreting cells.

I started as a postdoc in Sam Strober’s lab in Department of Medicine, at Stanford University Medical Center, in February 1976. Upstairs from his lab, in Len and Lee Herzenberg’s group at the Department of Genetics, there was intense research using one the first flow cytometry machines. I wished to determine if the IgG response obtained after LPS stimulation *in vitro* was derived from IgM-producing cells, i.e., if there was a true switching event occurring in the cultures. I sorted IgM and IgG positive and negative cells and cultured the populations separately, and could show that the IgG-producing cells came from IgM^+^, IgG^−^ precursors (4). One curious observation was that the main IgG subclasses produced were IgG2b and IgG3, and we did not detect very much IgG1, although the latter is the dominant subclass in serum.

Back in Stockholm a few years later, I read a manuscript by Peter Isakson et al. in Ellen Vitetta’s lab about a factor that induced an elevated IgG response, mostly of the IgG1 subclass, when given to LPS cultures. Their paper was published in 1982 in *J Exp Med* (5), in the same issue as a paper by Maureen Howard et al. in Bill Paul’s group about a factor that induced DNA synthesis in B cells together with anti-IgM (6). These two papers are to my knowledge the first to describe the function of what later became known as interleukin 4 (IL-4). Peter used supernatants from different T cell lines. We tested supernatants from primary mixed lymphocyte cultures that we obtained from Antonio Coutinho and they worked in a similar way, the elevated IgG being mostly IgG1. We were Susanne Bergstedt-Lindqvist, Paschalis Sideras, both PhD students and myself. Although they worked with me, neither were officially my students, because I only had a very small grant at the time.

It became clear that the stimuli dictated the subclass produced by B cells. The T-independent LPS gave rise to IgG2b and IgG3, but addition of factors produced by T cells caused B cells to produce IgG1 instead.

We realized eventually that we needed a more reliable source of the IgG1 induction factor, which was the name we gave it. For this reason, Paschalis and I and went to Lausanne to work in Marcus Nabholz’s lab at ISREC (Swiss Institute for Experimental Research). This was in the spring of 1983. In the neighboring lab Rob MacDonald and his group were working with continuous T cell lines from primary cultures. From the very back of the incubator, we retrieved a flask that appeared to have been forgotten. We re-stimulated the cells, cloned them and tested the supernatants for IgG1-inducing activity. There were several clones that were positive. Rob suggested later that the fact that the flask had been forgotten might have increased the chances to select for IL-4 secreting cells, due to autocrine activation. Usually, cells were re-stimulated with irradiated stimulatory cells plus IL-2, which most would probably have repressed IL-4 production.

We took the T cell clones with us back to Stockholm and decided to characterize the IgG1 induction factor in biochemical terms. For this, we developed a quantitative assay, using serial dilutions of the factor in cultures. We obtained this idea from Kendall Smith’s work with IL-2. We had met Ken at conferences and he was always very supportive of our work. We wrote a paper describing the different T cell clones, one of them being the 2.19 cell line, which we used later on to clone IL-4 (7). Another paper of ours described the biochemical characteristics of the IgG1 induction factor, which indicated that our factor had properties similar to Paul’s B-cell-stimulating factor (8). When Paschalis showed our data at a Keystone meeting, it did not attract very much attention. The general idea at the time was that each function was regulated by distinct factors and thus it was considered very unlikely that the same factor would induce proliferation together with anti-IgM and an elevated IgG1 response together with LPS.

In August 1983, I went to the Congress of Immunology in Kyoto. It was terribly hot and I was 7 months pregnant. The most exciting talks were those by Ellis Reinherz, Mark Davis, and James Allison, describing for the first time the T cell receptor. Also, I remember several people being very upset with the scientists who had claimed that T cells expressed immunoglobulin. That hypothesis died completely at this conference.

In connection with this meeting I visited Tasuku Honjo, who was then working at Osaka University. I had met him twice before in Sweden. He was well known for having correctly determined the gene order of the heavy-chain C gene segments in mice and for discovering the heavy-chain switch regions (9). I gave a seminar about our preliminary data with the IgG1 induction factor. Tasuku suggested collaboration: “I would like to clone switch factors” he said. Back in Sweden in December of 1983, I gave birth to my first child, John.

To attempt to clone the IgG1 induction factor without having purified it or a specific antibody was a bold attempt, which I had thought would not be within my reach. Tasuku, being a careful person and probably a bit suspicious of the cellular techniques, wanted first to test our assay to measure the response. So we sent him the protocol together with the necessary ingredients and they obtained the same response as we did. Paschalis and Susanne then started to collect 10^9^ cells of the 2.19 cell line to send to Honjo’s group. This took several months, since these were primary T cells stimulated by irradiated allogeneic spleen cells and they grew rather slowly. The final stimulation was done with concanavalin A (ConA). We sent the cells by the end of June 1984 and people in Honjo’s group prepared mRNA. A second batch of cells was sent later. At this time, we also received a lot of help from Lena Berggren (now Lena Ström), our technician. She eventually got a PhD with me as her supervisor and is now an established scientist working with genome stability and variation. Honjo’s group purified mRNA and as a first test, injected it into *Xenopus* oocytes and collected supernatants. The supernatants indeed gave an increased IgG1 response when given to LPS cultures. This result was obtained in October 1984. Then, Honjo’s coworkers transfected COS cells with a cDNA library from the 2.19 mRNA, took supernatants and sent them to us in Sweden. However, COS cell supernatants were inhibitory when given to LPS cultures, perhaps due to mycoplasma-infection, since this notoriously inhibits B cell activation *in vitro*. Tasuku was compelled to change strategy, and came up with a very ingenious one: They constructed an Sp6 expression vector and cloned cDNA out of 2.19 mRNA, did *in vitro* transcription, and injected the RNA into oocytes. The oocyte supernatants were then sent to us for testing. This strategy worked well and we could detect IgG1-inducing activity for as many as 45,000 mixed clones. The numbers of clones per batch decreased for each round of testing, until we finally had single positive clones. We reached single clones in the summer of 1985. The cDNA from a positive clone was sequenced and found to encode a protein of MW around 15,000. It had some homologies suggesting a distant relation to γ interferon (γIFN) and granulocyte macrophage colony-stimulating factor (GM-CSF). We tested the recombinant supernatant and found that it induced higher MCH class II levels and increased DNA synthesis when given together with anti-IgM. We concluded that the IgG1 induction factor and the B-cell-stimulating factor-1 (BSF-1) were products of the same gene. First, we wrote two papers (letters) that we submitted to Nature. One, for which Yoshihiko Noma was first author and Tasuku was senior author, dealt with the molecular cloning. The other, for which Paschalis was first author and I was senior author, was about the function of the cloned factor on B cell responses. The editors of Nature wished us to merge the two papers and invited us to write an article instead. This we did, and the paper was published in February 1986 (1). In April the same year, Lee et al. published a paper identifying the same cytokine, also by cDNA cloning. The cytokine could activate T and B cells, as well as mast cells. It acted as a B-cell-stimulating factor, and induced IgG1 and IgE (10). At the end of both of the papers, we proposed that the cytokine should be called “interleukin-4.” Our paper attracted a lot of attention, partly because the cloning method was unique. It should be pointed out that cDNA cloning at this time was not a technique that any lab could do. In fact, only a few groups in the world mastered it, and Honjo’s lab was one of the best. Secondly, the two papers revealed that IL-4 was very pleiotropic, having effects on at least three cell types and inducing different responses in B cells, as opposed to other known cytokines, and quite different from the idea that one cytokine would only activate a single response. The molecular cloning of IL-4 inspired a great deal of further research. In August the same year, a paper was published about cloning of the cDNA that encoded human IL-4 (11). Soon it became apparent that both human and mouse IL-4 induced an IgE response (10, 12) and it was subsequently showed, using the knockout technology, that IL-4 is essential for IgE expression in mice (13). Thus, IL-4 was found to be clinically relevant for understanding the induction of atopic allergy. Furthermore, the finding that IL-4 is secreted by a subpopulation of T helper cells, called TH2 (14), inspired a flood of research in humans. In the field of Ig class switching, IL-4 is still one of the model stimuli in research of class switch recombination in the mouse, because of its efficient induction of IgG1 and because the discovery that stimuli direct switching by inducing germ-line transcripts (15, 16). Many cytokines have been described since then: a glance at internet reveal the existence of IL-38.

## Conflict of Interest Statement

The author declares that the research was conducted in the absence of any commercial or financial relationships that could be construed as a potential conflict of interest.
